# The Serine/Threonine Phosphatase PP4 Is Required for Pro-B Cell Development through Its Promotion of Immunoglobulin VDJ Recombination

**DOI:** 10.1371/journal.pone.0068804

**Published:** 2013-07-16

**Authors:** Yu-wen Su, Ya-ping Chen, Ming-yu Chen, Michael Reth, Tse-Hua Tan

**Affiliations:** 1 Immunology Research Center, National Health Research Institutes, Zhunan, Miaoli County, Taiwan; 2 Center for Biological Signalling Studies BIOSS and Faculty of Biology, University of Freiburg, Max Planck Institute of Immunobiology and Epigenetics, Freiburg, Germany; 3 Department of Pathology & Immunology, Baylor College of Medicine, Houston, Texas, United States of America; Institut Pasteur, France

## Abstract

PP4 phosphatase regulates a number of crucial processes but the role of PP4 in B cells has never been reported. We generated B cell-specific *pp4* knockout mice and have identified an essential role for PP4 in B cell development. Deficiency of PP4 in B lineage cells leads to a strong reduction in pre-B cell numbers, an absence in immature B cells, and a complete loss of mature B cells. In PP4-deficient pro-B cells, immunoglobulin (Ig) DJ_H_ recombination is impaired and Ig µ heavy chain expression is greatly decreased. In addition, PP4-deficient pro-B cells show an increase of DNA double-strand breaks at Ig loci. Consistent with their reduced numbers, residual PP4-deficient pre-B cells accumulate in the G1 phase, exhibit excessive DNA damage, and undergo increased apoptosis. Overexpression of transgenic Ig in PP4-deficient mice rescues the defect in B cell development such that the animals have normal numbers of IgM^+^ B cells. Our study therefore reveals a novel function for PP4 in pro-B cell development through its promotion of V_H_DJ_H_ recombination.

## Introduction

B cell development initiates in the bone marrow (BM) of adult mice and is a tightly controlled process. Developing B cells can be divided chronologically into six Hardy fractions (Frs.) A to F according to the recombination status of the immunoglobulin (Ig) heavy chain (HC) locus, the Ig light chain (LC) locus, and the expression pattern of particular cell surface markers [1], [2]. The process starts with D-J_H_ recombination in Fr. A cells, followed by V_H_-DJ_H_ recombination in Fr. B and Fr. C cells [2], [3]. When a differentiating B cell reaches the Fr. D stage, V_L_-J_L_ recombination commences [2]. Successful Ig V_H_DJ_H_/V_L_J_L_ recombination leads to the expression of a surface IgM-containing BCR complex that enables a B cell to continue to the Fr. E and Fr. F stages [4]. At the molecular level, DJ_H_/V_H_DJ_H_ recombination is initiated when two Ig gene segments flanked by recombination signal sequences (RSSs) are paired and cleaved by RAG [5], [6]. The two gene segments are brought together by the cell’s non-homologous end joining (NHEJ) machinery via the sequential recruitment of NHEJ factors. A deficiency of any of these factors results in a failure in DJ_H_/V_H_DJ_H_ recombination, an early block in B cell development, and ultimately a shortage of mature B lymphocytes [7].

Protein phosphatase 4 (PP4) belongs to the type 2A protein serine/threonine phosphatase (PP2A) family. In mammals, the catalytic subunit of PP4 (PP4c) selectively binds to one or two of several different regulatory subunits, including R1 [8], [9], R2 [10], R3 [11], R4 [12], α4 [13], [14], TIP [15], TIPRL [16], and Smek [17], to form a PP4 holoenzyme. The composition of the PP4 holoenzyme presumably determines its catalytic activity and also confers its substrate and tissue specificity [18]. At the cellular level, PP4 activity is required for microtubule organization and centrosome maturation via mechanisms that are highly conserved among mammalian species [19], [20], [21]. PP4 is also necessary for DNA repair via the homologous recombination pathway through dephosphorylation of the RPA2 subunit of replication protein A [22], and through dephosphorylation of γH2AX during cell division [18], [23], [24]. Lastly, PP4 has been implicated in multiple signal transduction pathways, including pre-TCR/TCR signaling [25], TNF-α signaling [26], [27], Toll-like receptor 4 signaling [28], and NF-κB signaling [29], [30].

T cell-specific deletion of PP4 in mice leads to a partial block in thymocyte development at the double negative (DN) stage. The Ca^2+^ mobilization and PLC-γ1 phosphorylation normally induced by anti-CD3 stimulation are impaired in these PP4-deficient cells [25]. Whether PP4 plays an analogous role in B cell development is unknown. In this study, we utilized mb-1/cre mice to delete the *pp4c* gene specifically in B cells and identified a pivotal role for PP4 in pro-B cell development. Deletion of PP4 severely disrupted pro-B cell differentiation and consequently led to a complete absence of mature B cells. In PP4-deficient pro-B cells, DJ_H_ recombination was greatly reduced and Ig µ HC expression was decreased. We also found that PP4-deficient pre-B cells *in vivo* accumulated in the G1 phase, showed an elevated level of DNA damage, and underwent increased apoptosis. Significantly, PP4-deficient pro-B cells transgenically expressing IgM successfully differentiated into normal numbers of IgM^+^ B cells. Our results therefore reveal the indispensable role of PP4 in promoting the V_H_DJ_H_ recombination required for continued pro-B cell differentiation and the production of mature B cells.

## Materials and Methods

### Mice

PP4C^F/F^ mice [25], mb-1/cre mice [31], and Ig^HEL^ transgenic mice [32] generated as previously described were maintained in strict accordance with the recommendations of the Guide for the Care and Use of Laboratory Animals of the National Health Research Institutes (NHRI). The Ig^HEL^ transgenic and mb-1/cre mice used in all experiments were heterozygous. All protocols were approved by NHRI’s Institutional Animal Care and Use Committee (Permit Number: 099111-A), and all efforts were made to minimize suffering.

### BrdU Incorporation *in vivo* and *in vitro*

For *in vivo* BrdU incorporation, mice were injected i.p. with 2 mg BrdU (Simga) in 200 µl sterile PBS at 16 h and 24 h before sacrifice and cell sorting. For *in vitro* BrdU incorporation, isolated cells were cultured for 16 h in RPMI 1640 medium containing 10% FCS (Hyclone) and 10 µM BrdU prior to analysis using a BrdU-Flow kit (BD Pharmingen™).

### Cell Sorting and Cell Culture

Single-cell suspensions were prepared from the BM of mice by gentle pipetting and sieving through 70-µm nylon mesh filters (Falcon; BD). BM cells were cultured in RPMI 1640 medium containing 10% FCS and 2 mM L-glutamine (Invitrogen), 100 U penicillin-streptomycin (Invitrogen), 100 mM Hepes (Sigma-Aldrich), and 0.055 mM 2-mercaptoethanol (Gibco). For flow cytometric analyses, cells were suspended in FACS buffer (2% BSA in PBS containing 0.1% NaN3).

### Comet Assays

To perform comet assays to detect DNA damage, Fr. B and Fr. C cells were sorted by FACSAria (BD) and processed using the OxiSelect™ Comet Assay Kit following the manufacturer’s instructions (Cell Biolabs). Data were analyzed using Comet Assay IV™ v4.3 software.

### Confocal Imaging

Fr. B and Fr. C cells were sorted, fixed in 4% paraformaldehyde, and permeabilized in 0.2% saponin. After blocking with Image-iT™ FX signal enhancer (Invitrogen), cells were stained with anti-mouse IgM-FITC (HC-specific) antibody (Ab) (BD), seeded on Superfrost® Plus microscope slides (Thermo Scientific), and mounted using Prolong® Gold anti-fade reagent plus DAPI (Invitrogen).

### Ig DJ_H_ Recombination Assay and Coding End Assay

For D_H_-J_H_ recombination analysis, genomic DNA from 5×10^5^ cells was extracted and subjected to nested PCR utilizing a primer 5′ of the DFL16.1 segment and a primer 3′ of the J_H_4 segment in the Ig HC locus (Fig. S1). 1 ng of genomic DNA was utilized for first-round PCR using the following primers:

dfl16.1∶5′-CCAGGGCTTTTTGTGAAGGGATCTACTACTGT-3′ and jh4∶5′-TTCTTCAAATGAGCCTCCAAAGTCC-3′. PCR conditions were as follows: 96°C for 3 min; 2 cycles of 94°C for 40 sec, 65°C for 45 sec, and 72°C for 1 min 45 sec; 3 cycles of 94°C, 63°C and 72°C for these same time intervals; and 18 cycles of 94°C, 60°C and 72°C for these same time intervals. For second-round PCR, primers in-dfl16.1∶5′-AAGGGATCTACTACTGTGTTTATTACTACGGTAGTAGCTAC-3′ and in-jh4∶5′-AGGCTCTGAGATCCCTAGACAG-3′ were used with PCR conditions as follows: 94°C for 3 min; 3 cycles of 94°C, 67°C and 72°C for the same time intervals as used for first-round PCR; and 24 cycles of 94°C, 64°C and 72°C for these same time intervals. PCR products were separated by 1% agarose gel electrophoresis and capillary-transferred to nylon membranes. Blots were hybridized with 5 ng/ml DIG-labeled PCR probe synthesized with the following primers: j4p: 5′-ATTACTATGCTATGGACTACTGGGGTCAAGG-3′ and in-jh4∶5′-CAGTAATACATGGCCGTGTCCTCATACC-3′, and detected using the DIG system following the manufacturer’s instructions (Roche). To detect DNA double-strand breaks at Ig loci, oligonucleotides BW-1∶5′-GCGGTGACCCGGGAGATCTGAATTC-3′ and BW-2∶5′-GAATTCAGATC-3′ were annealed for the preparation of BW linker as described previously [33]. 1 µg of genomic DNA from sorted Fr. B cells was treated with 0.1 U/50 µl of Klenow at 25°C for 30 min. After Klenow-inactivation, genomic DNA was washed and ligated with 1000 pM/50 µl of BW linker overnight. 500 ng of ligated DNA was utilized for first-round PCR using the primer 16.1b: 5′-GCCTTCCACAAGAGGAGAAG-3′ at the upstream of DFL16.1 segment and the primer BW-1 (Fig. S2). PCR conditions were as follows: 96°C for 3 min; 3 cycles of 94°C for 1 min, 58°C for 2 min, and 72°C for 1 min 45 sec; 12 cycles of 94°C for 1 min, 62°C for 2 min, and 72°C for 1 min 45 sec. For second-round PCR, the primer 16.1a: 5′-GAAGTCCCCCAGAGACAGAC-3′ at the downstream of primer 16.1b and BM-1 were utilized. PCR conditions were as follows: 94°C for 3 min; 27 cycles of 94°C for 1 min, 62°C for 2 min and 72°C for 1 min. PCR products were separated by 1% agarose gel electrophoresis and capillary-transferred to nylon membranes. Blots were hybridized with 700 ng/ml DIG-labeled PCR probe of 220 bp synthesized with primers 16.1a and COD-R: 5′- CCAGGCAGCACGGTTGAGTTT-3′, and detected as described above.

### Flow Cytometry

Single-cell suspensions of 1×10^6^ cells were washed twice with FACS buffer and maintained in the dark at 4°C throughout the experiment. A CantoII flow cytometer (BD) and FACSDiva software (BD) were used for the acquisition of flow cytometric data, and FlowJo software (Tree Star, Inc.) was used for analysis. For apoptosis assays, cells were stained with B220-APC and Annexin V-FITC using the Apoptosis Detection Kit I (BD). To stain and to sort B cells, BM cells were incubated on ice for 15 min with anti-mouse Abs as follows: BP-1-FITC/PE, IgD-Pacific Blue™ (both from Biolegend); IgM-FITC/eFluor® 450 (eBioscience), B220-PE/PerCP/APC-Cy7, CD21-PE, CD5-PE, CD43-APC, CD23-PE-Cy7, CD24-PE-Cy7, and IgM-APC/PE-Cy7 (all from BD). Immunostained cells were washed twice in ice-cold FACS buffer prior to sorting by FACSAria cell sorter (BD). For intracellular staining, cells were fixed and permeabilized using the BrdU-Flow kit (BD Pharmingen™) prior to incubation with µ HC-FITC or BrdU-Alexa Fluor® 647 (all from BD).

### Statistical Analysis

Data were analyzed by a one-tailed distribution, type 3 Student’s t-test. Differences between treatment groups with *p*-values ≤0.05 were considered statistically significant.

## Results

### PP4 Deficiency Leads to a Severe Block in B Cell Development

**Figure 1:**
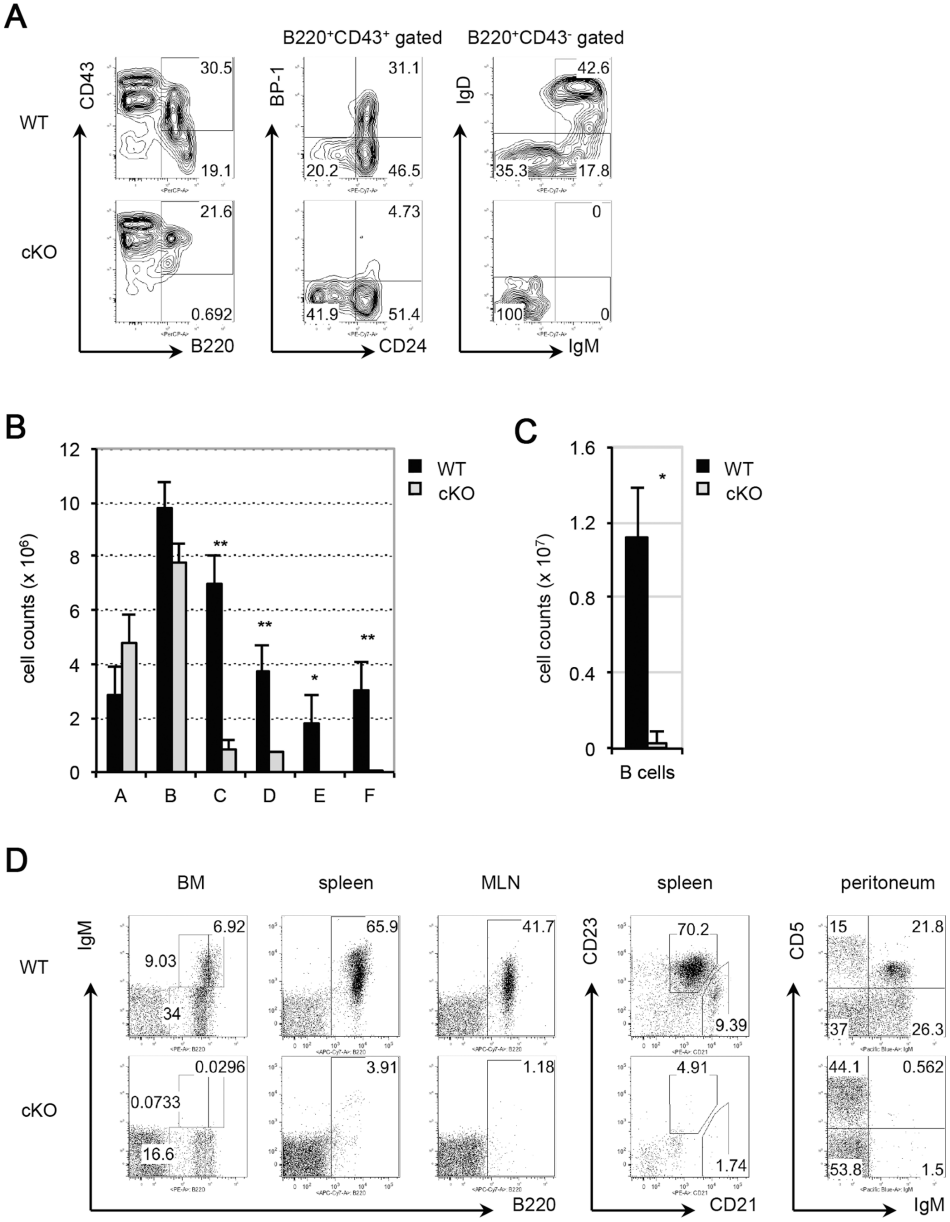
PP4 deficiency induces a severe block in B cell development. (A) Flow cytometric profiles of: total BM lymphocytes from WT and cKO mice analyzed for B220 vs CD43 expression (left panel); B220^+^CD43^+^ BM lymphocytes analyzed for CD24 vs BP-1 (middle panel); and B220^+^CD43^−^ BM lymphocytes analyzed for IgM vs IgD (right panel). (B) Quantitative cell count analysis of Frs. A to F cells from WT and cKO mice. (C) Quantitative cell count analysis of: splenic B220^+^ B cells from WT and cKO mice. (D) Flow cytometric profiles of BM, spleen and MLN lymphocytes from WT and cKO mice (n = 4/group) analyzed for B220 vs IgM; B220^+^ splenocytes analyzed for CD21 vs CD23; and peritoneal lymphocytes analyzed for IgM vs CD5, as indicated. For A-D, results are representative of three independent experiments. For B and C, data are the mean ± SD (n = 4) (*p≤0.05; **p≤0.005).

To study the role of PP4 in B cell development, mb-1/cre mice [31] were bred with *pp4c loxp*-flanked (*floxed*) mice [25] to generate mb-1/cre;PP4^F/F^ (designated here as cKO) mice. As a control, mb-1/cre mice were bred with *pp4c* WT mice to obtain mb-1/cre;PP4^+/+^ (designated here as WT) mice. B cell subsets, including Fr. A (B220^+^CD43^+^CD24^−^BP-1^−^; pre-pro-B cells), Fr. B (B220^+^CD43^+^CD24^+^ BP-1^−^; pro-B cells), Fr. C (B220^+^CD43^+^CD24^+^BP-1^+^; pro-B plus large pre-B cells), Fr. D (B220^+^CD43^−^IgM^−^IgD^−^; small pre-B cells), Fr. E (B220^+^CD43^−^IgM^+^IgD^−^; immature B cells), and Fr. F (B220^high^CD43^−^IgM^low^IgD^high^; mature B cells), from the BM of WT and cKO mice were analyzed by FACS. In cKO mice, combined staining with anti-B220 and anti-CD43 Abs revealed that the B220^+^CD43^−^ BM lymphocyte population containing Frs. D to F was greatly reduced (Fig. 1A, left panel). Gating of B220^+^CD43^+^ BM lymphocytes on CD24 plus BP-1 expression showed that the percentage of CD24^+^BP-1^+^ cells (Fr. C) was strongly reduced in the mutant compared to the control (WT, 31.1% vs cKO, 4.7%) (Fig. 1A, middle panel). Among the residual B220^+^CD43^−^ BM lymphocytes in cKO mice, only IgM^−^IgD^−^ cells (Fr. D) were present (Fig. 1A, right panel). Cell count analyses revealed severe reductions in the Fr. C and Fr. D populations in cKO mice, and the complete absence of the Fr. E and Fr. F subsets (Fig. 1B, Table 1). In the spleen and mesenteric lymph nodes (MLN) of cKO mice, mature B cells were barely detectable (Fig. 1C, 1D, Table 2). In addition, CD5^+^IgM^+^ B1a B cells were missing from the peritoneum (Fig. 1D). Thus, B cell development in cKO mice is severely blocked before the Fr. C stage, leading to the complete loss of immature and mature B cells.

**Table 1:** Summary of cell counts (x10^6^) of B cell fractions in WT and cKO mice.

Mice	Total BM	Fr. A	Fr. B	Fr. C	Fr. D	Fr. E	Fr. F
WT	57.9±10.63	2.89±0.39	9.75±3.68	6.98±1.79	2.96±1.75	1.44±1.17	2.43±1.62
cKO	55.2±1.65	4.76±1.11	7.76±0.71	0.87±0.28	0.71±0.05	0±0	0±0
*P*	0.2992	0.0190	0.1484	0.0006	0.0014	0.0171	0.0049

Cell counts of total BM and Frs. A to F cells from WT and cKO mice (n = 4/group) were calculated following immunostaining to detect the appropriate markers and analysis by flow cytometry. Results are representative of three independent experiments.

**Table 2:** Cell counts (×10^7^) of total splenocytes and splenic B cells in WT and cKO mice.

Mice	Splenocytes	Splenic B cells
WT	4.54±0.56	1.11±0.26
cKO	0.50±0.13	0.03±0.05
*p*	0.0024	0.0085

Total cell counts of splenocytes and splenic B cells (B220^+^ gated lymphocytes) from WT and cKO mice (n = 4/group) were calculated following immunostaining to detect the appropriate markers and analysis by flow cytometry. Results are representative of three independent experiments.

**Figure 2:**
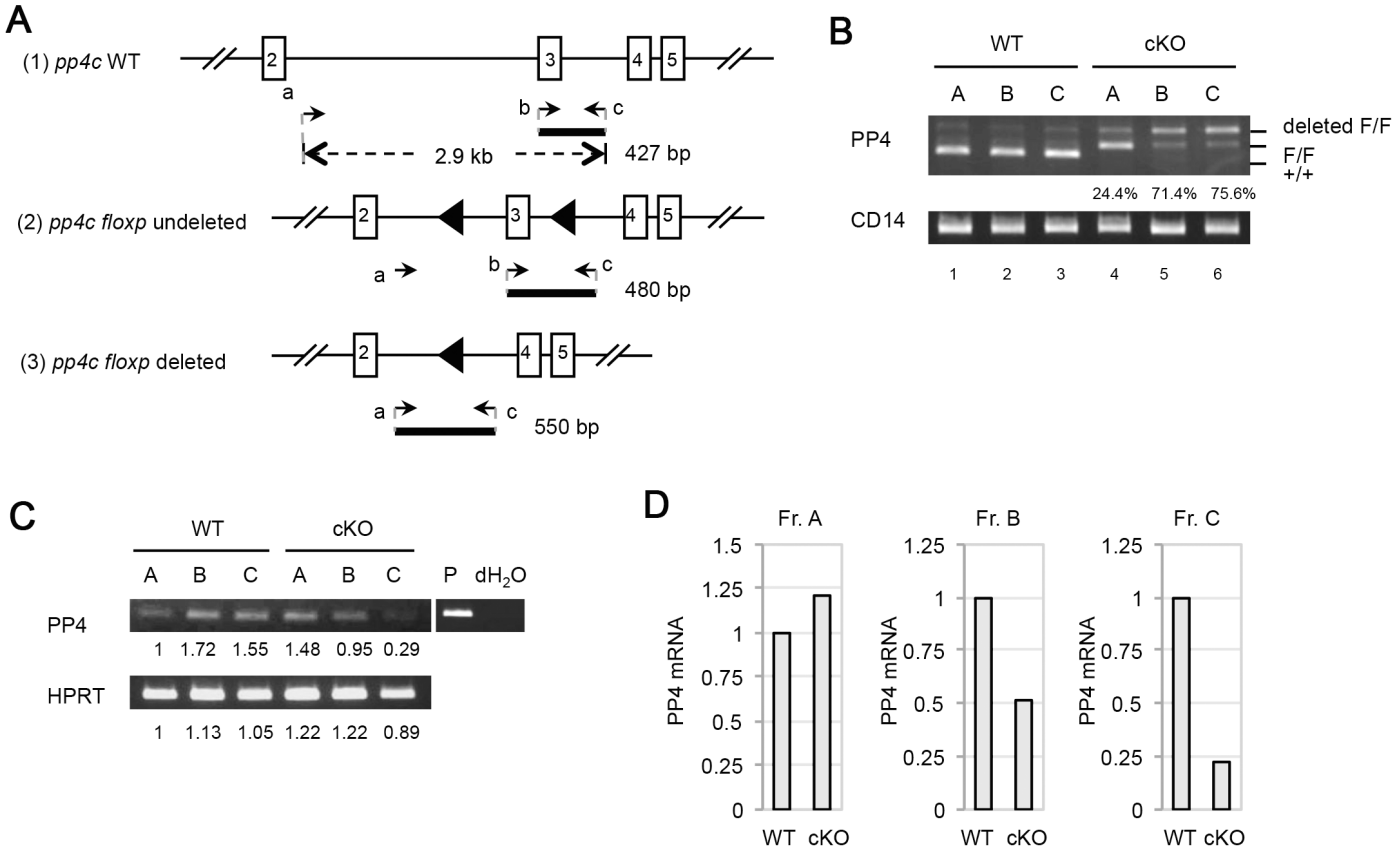
Efficiency of *pp4c* deletion in B cells. (A) Schematic illustration of the targeting strategy used to delete *pp4c* and the primers designed to detect deletion efficiency. For genomic PCR, forward primers “a” (5′-ACGTGATTTGCGAAAGCCTCTCA-3′) and “b” (5′-CTTGGTAGAAGAGAGCAACGTGCAG-3′), and reverse primer “c” (5′-TGCTCTGGTGGCAGGAGATGTGTG-3′), were used as indicated. The PCR products of the WT *pp4c* allele (427 bp; 1); the *pp4c floxp* allele before cre-mediated deletion (480 bp; 2); and the *pp4c floxp* allele after cre-mediated deletion (550 bp; 3) are shown. (B) PCR analysis of genomic DNA from Frs. A, B and C cells from WT and cKO mice (n = 1/group) showing products representing the WT *pp4c* allele (+/+), the *pp4c* floxp allele (F/F), and the deleted *pp4c* allele (deleted F/F). Percentages shown are deletion efficiencies. CD14, loading control. Results are representative of three independent experiments. (C) RT-PCR analysis of PP4 and HPRT mRNAs in Frs. A, B and C cells from WT and cKO mice (n = 2/group). Numbers are the relative mRNA levels quantified by Image J and normalized to the WT Fr. A value (set to 1). P, positive control for PP4. HPRT, loading control. Results are representative of three independent experiments. (D) Quantitation of the mRNA levels in the cells in (C) after normalization to HPRT values.

To determine the deletion efficiency mediated by mb-1/cre, genomic DNA samples from Frs. A–C cells sorted from WT and cKO mice were subjected to PCR analysis. A scheme illustrating the primers used to detect *pp4c* deletion efficiency is shown in Fig. 2A. In Frs. A–C from cKO mice, *pp4c* deletion efficiency was 24.4%, 71.4%, and 75.6%, respectively (Fig. 2B, *lanes* 4–6). In parallel to our deletion efficiency determinations, we extracted total RNA from WT and cKO Frs. A-C cells to analyze PP4 mRNA by RT-PCR (Fig. 2C). After normalization to HPRT expression, PP4 mRNA from cKO mice was reduced by 50% in Fr. B cells and by 77% in Fr. C cells compared to WT controls (Fig. 2D). Thus, consistent with a previous report [34], our results show that mb-1/cre-mediated pp4c gene deletion initiates in Fr. A cells, with higher deletion efficiencies occurring in Fr. B and Fr. C cells. The finding suggests that the developmental block at Fr. C stage in cKO mice likely correlates with the higher *pp4c* deletion efficiency in this population.

### Impaired DJ_H_ Recombination and Increased DNA Double-strand Breaks at Ig loci in PP4-deficient Pro-B Cells

**Figure 3:**
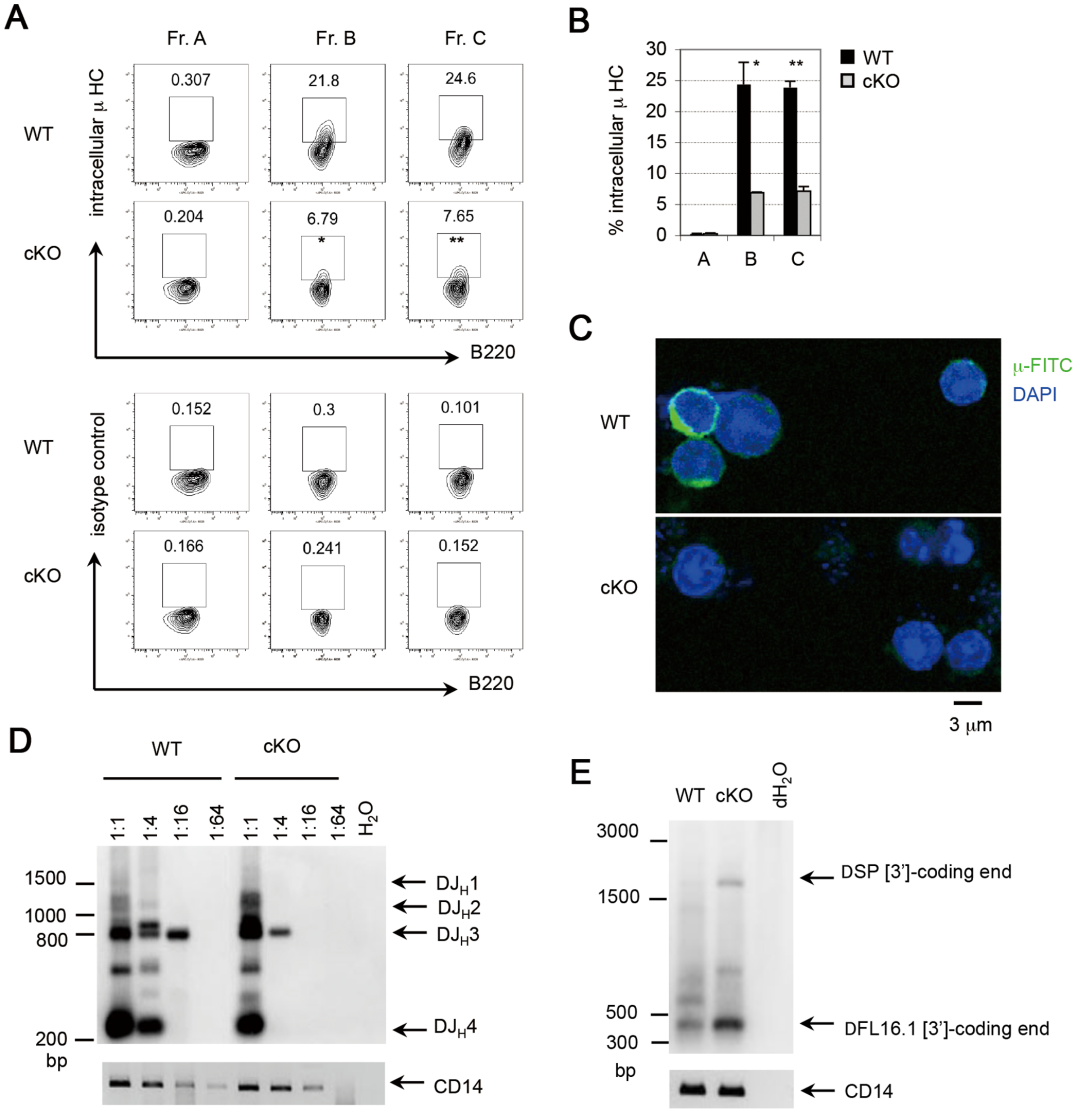
Reduced Ig µ HC expression and impaired DJ_H_ recombination in PP4-deficient pro-B cells. (A) Flow cytometric profiles of Frs. A, B and C cells from WT and cKO mice (n = 3/group) analyzed for B220 vs intracellular µ HC (upper panel), or B220 vs isotype control (lower panel). Results are representative of two independent experiments. (B) Quantitation of percentages of the Fr. A, B and C cells in (A) expressing intracellular µ HC. Data are the mean ± SD (n = 2). (C) Confocal images of intracellular µ HC staining in Fr. B cells from WT and cKO mice (n = 2/group). DAPI, nuclei. Results are representative of three independent experiments. (D) Southern blot of genomic DNA from Fr. B cells of WT and cKO mice (n = 2/group) subjected to a DJ_H_ recombination assay. Genomic DNA was serially diluted from 1∶1 to 1∶64 prior to nested PCR. Bands were visualized using DIG-labeled southern blotting. CD14, loading control. Results are representative of two independent experiments. (E) Southern blot of genomic DNA from Fr. B cells of WT and cKO mice subjected to the coding end assay. Genomic DNA was filled by Klenow, ligated with double-strand oligo linker of 5′-overhang, and subjected to nested PCR. Bands were visualized using DIG-labeled southern blotting. CD14, loading control. Ten mice per group were used in this experiment, and two independent experiments were performed.

To further characterize the effects of PP4 deficiency during early B cell development, Frs. A–C cells from WT and cKO mice were subjected to intracellular staining to detect Ig µ HC. In WT mice, Ig µ^+^ cells constituted 0.3%, 21.8%, and 24.6% of Frs. A, B and C cells, respectively, whereas the corresponding percentages in cKO mice were 0.2%, 6.7%, and 7.6% (Fig. 3A, B). Thus, Ig µ HC expression was strongly reduced in the absence of PP4 beginning at the Fr. B stage, an observation confirmed using confocal microscopy to examine Fr. B cells from WT and cKO mice (Fig. 3C). To assess whether this reduction in Ig µ HC was due to impaired Ig DJH recombination, we subjected the genomic DNA of WT and cKO Fr. B cells to an *in vitro* DJH recombination assay. In both WT and cKO Fr. B cells, PCR products representing rearranged DJ_H_1, DJ_H_2, DJ_H_3 and DJ_H_4, gene segments appeared when the template was diluted 1∶1 (Fig. 3D). In WT mice, the rearranged DJH3 gene segment remained detectable until the template dilution reached 1∶16. In contrast, the rearranged DJ_H_3 gene segment was detectable up to a dilution of only 1∶4 in cKO Fr. B cells, suggesting that PP4 deficiency inhibits D-J_H_ recombination. These data imply that PP4 is involved in the regulation of Ig DJ_H_/V_H_DJ_H_ recombination such that loss of *pp4c* impairs Ig µ HC expression.

During VDJ recombination, the hairpin coding ends are generated after the cleavage of recombination signal sequences (RSSs). Before the rejoining process is initiated, hairpin coding ends are opened to form double-strand ends [35]. To address whether the impaired Ig DJ_H_ recombination induced by PP4 deficiency is associated with double-strand breaks (DSBs) at RSSs, the genomic DNA of WT and cKO Fr. B cells was extracted and then subjected to coding end assay as described previously [33], [36]. The PCR product of approximate 350 bp, indicating the DSB at the 3′ RSS of DFL16.1 segment (DFL16.1 [3′]), appeared in both WT and cKO Fr. B cells (Fig. 3E and Fig. S2). However, the formation of DFL16.1 [3′]-coding end in cKO mice was nearly threefold more than that in WT mice. In addition, the PCR product of approximate 2 kb, indicating the DSB at the 3′ RSS of DSP segment (DSP [3′]), appeared in cKO mice. By contrast, DSP [3′]-coding end in WT mice was barely detectable. These data suggest that PP4 is involved in the regulation of coding end joining during Ig DJ_H_/V_H_DJ_H_ recombination such that loss of *pp4c* causes DNA DSBs at Ig loci.

### PP4 Deficiency is Associated with DNA Breaks and Impaired B Cell Homeostasis

**Figure 4:**
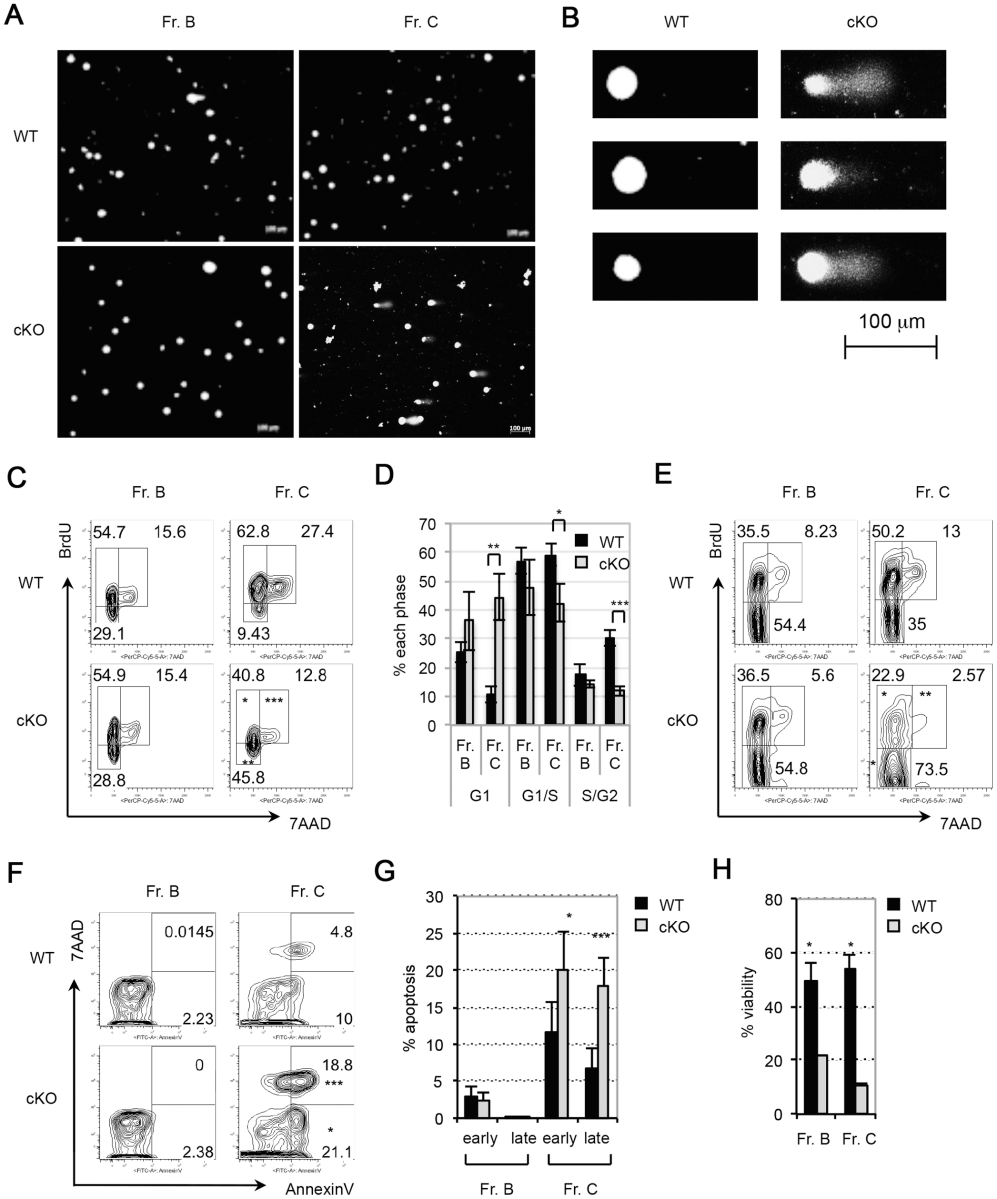
PP4 deficiency causes excessive DNA damage and impairs B cell homeostasis. (A) Microscopic images of comet assays of Fr. B and Fr. C cells from WT and cKO mice (n = 2/group). Results are representative of two independent experiments. (B) Enlarged microscopic images of the comet assays of the WT and cKO Fr. C cells in (A). (C) *In vivo* BrdU incorporation assay. WT and cKO mice (n = 3/group) were i.p. injected with BrdU at 16 h and 24 h before isolation of Fr. B and Fr. C cells by cell sorting. Cells were subjected to intracellular staining with 7AAD plus anti-BrdU and analyzed by flow cytometry. Numbers in quadrants represent the percentage of the indicated population relative to the total. Results are representative of three independent experiments. (D) Quantitation of the percentages of G1, G1/S, and S/G2 phase cells among the Fr. B and Fr. C cells in (C). Data are the mean ± SD (n = 3). (E) *In vitro* BrdU incorporation assay. Fr. B and Fr. C cells were sorted from WT and cKO mice (n = 2/group), cultured for 16 h in the presence of BrdU, and subjected to intracellular staining and flow cytometric analysis as for (C). Results are representative of two independent experiments. (F) *In vivo* apoptosis assay. Fr. B and Fr. C cells from WT and cKO mice (n = 4/group) were stained to detect AnnexinV plus 7AAD and analyzed by flow cytometry. Numbers in the lower right quadrant indicate the percentage of cells in early apoptosis, whereas the values in the upper right quadrant indicate the percentage of cells in late apoptosis. Results are representative of two independent experiments. (G) Quantitation of Fr. B and Fr. C cells in early or late apoptosis as calculated from the results in (F). Data are the mean ± SD (n = 4–6/group). (H) *In vitro* viability assay. Fr. B and Fr. cells were sorted from WT and cKO mice (n = 2/group) and cultured for 16 h under standard conditions. Cells were surface-stained with 7AAD and the percentage of viable cells was calculated. Data are the mean ± SD (n = 2). Results are representative of two independent experiments (*p≤0.05, **p≤0.005, ***p≤0.0005).

During Ig gene rearrangement, DNA breaks are repaired mainly by factors in the NHEJ pathway [37]. The impaired Ig DJ_H_/V_H_DJ_H_ recombination by PP4 deficiency raised the possibility that this phenomenon was associated with excessive DNA damage. To address this question, WT and cKO Fr. B and Fr. C cells were subjected to comet assays in which the extent of DNA damage was determined by measuring the percentage of comet tail intensity and the ratio of comet tail length relative to head length. The degree of DNA damage was similar in WT and cKO Fr. B cells (Fig. 4A and Table 3). However, the percentage of tail intensity and the tail length ratio in cKO Fr. C cells were both increased compared to WT Fr. C cell values (Fig. 4A, 4B and Table 3), suggesting that DNA damage is more severe in Fr. C cells lacking PP4.

**Table 3:** Comet scores of Fr. B and Fr. C cells from WT and cKO mice.

Frs.	Mice	Tail % intensity	Tail length/head length
Fr. B	WT (n = 36)	13.82±14.62	1.00±0.31
	cKO (n = 57)	10.33±9.66	1.03±0.38
	*P*	0.1082	0.3495
Fr. C	WT (n = 61)	10.79±12.97	0.97±0.85
	cKO (n = 41)	16.50±15.43	1.84±1.25
	*p*	0.0275	0.0001

Tail % intensity, tail intensity expressed as a percentage of the comet’s total intensity; tail length, the horizontal distance from the centre of the head to the end of the tail; head length, the horizontal distance from the start of the head to the end of the head. Two mice per group were used in this experiment, and two independent experiments were performed.

To understand the effect of PP4 deficiency on cell-cycle progression *in vivo*, WT and cKO mice were injected with BrdU, and Fr. B and Fr. C cells were isolated and subjected to cell-cycle analysis. Cells of the BrdU^−^7AAD^−^, BrdU^+^7AAD^−^, BrdU^+^7AAD^+^, and BrdU^−^7AAD^+^ populations were defined as G1, G1/S, S/G2, and G2/M phase cells, respectively. We found that the percentages of Fr. B cells in each phase were comparable between WT and cKO mice (Fig. 4C). However, in cKO Fr. C cells, a 33% increase in G1 phase cells and a corresponding reduction in cells in the G1/S plus S/G2 phases were observed (Fig. 4C, 4D). To confirm these results *in vitro*, we cultured WT and cKO Fr. B and Fr. C cells for 16 h in the presence of BrdU and analyzed its incorporation. Similar to our *in vivo* results, no significant difference in the cell-cycle was observed between WT and cKO Fr. B cells (Fig. 4E). However, compared to WT Fr. C cells, cKO Fr. C cells showed a 2-fold increase in the percentage of G1 phase cells, while the percentages of G1/S and S/G2 phase cells were correspondingly diminished (Fig. 4E). Thus, PP4 deficiency affects the cell-cycle progression of Fr. C cells, leading to an accumulation of G1 phase cells and a reduction in S phase cells.

To investigate if PP4 deficiency was associated with increased apoptosis, freshly sorted WT and cKO Fr. B and Fr. C cells were subjected to AnnexinV plus 7AAD staining. While percentages of early apoptotic (AnnexinV^+^7AAD^−^) and late apoptotic (AnnexinV^+^7AAD^+^) cells were comparable between WT and cKO Fr. B cells, significant increases in early and late apoptotic cells were observed in cKO Fr. C cells compared to WT controls (Fig. 4F, 4G). To confirm this effect on cell viability *in vitro*, WT and cKO Fr. B and Fr. C cells were cultured in RPMI 1640 medium containing 10% FCS for 16 h followed by 7AAD staining. The percentage of viable cells in the cKO Fr. B culture was drastically reduced compared to the control (WT, 49.4% vs. cKO, 21.7%), as was true for the cKO Fr. C culture (WT, 53.7% vs. cKO, 10.6%) (Fig. 4H). Taken together, these analyses indicate that PP4 deficiency severely interferes with the cell-cycle of Fr. C cells and induces their apoptosis, thereby disrupting B cell homeostasis.

### Rescue of the Development of PP4-deficient Pro-B Cells by Ig Transgene Expression

**Figure 5:**
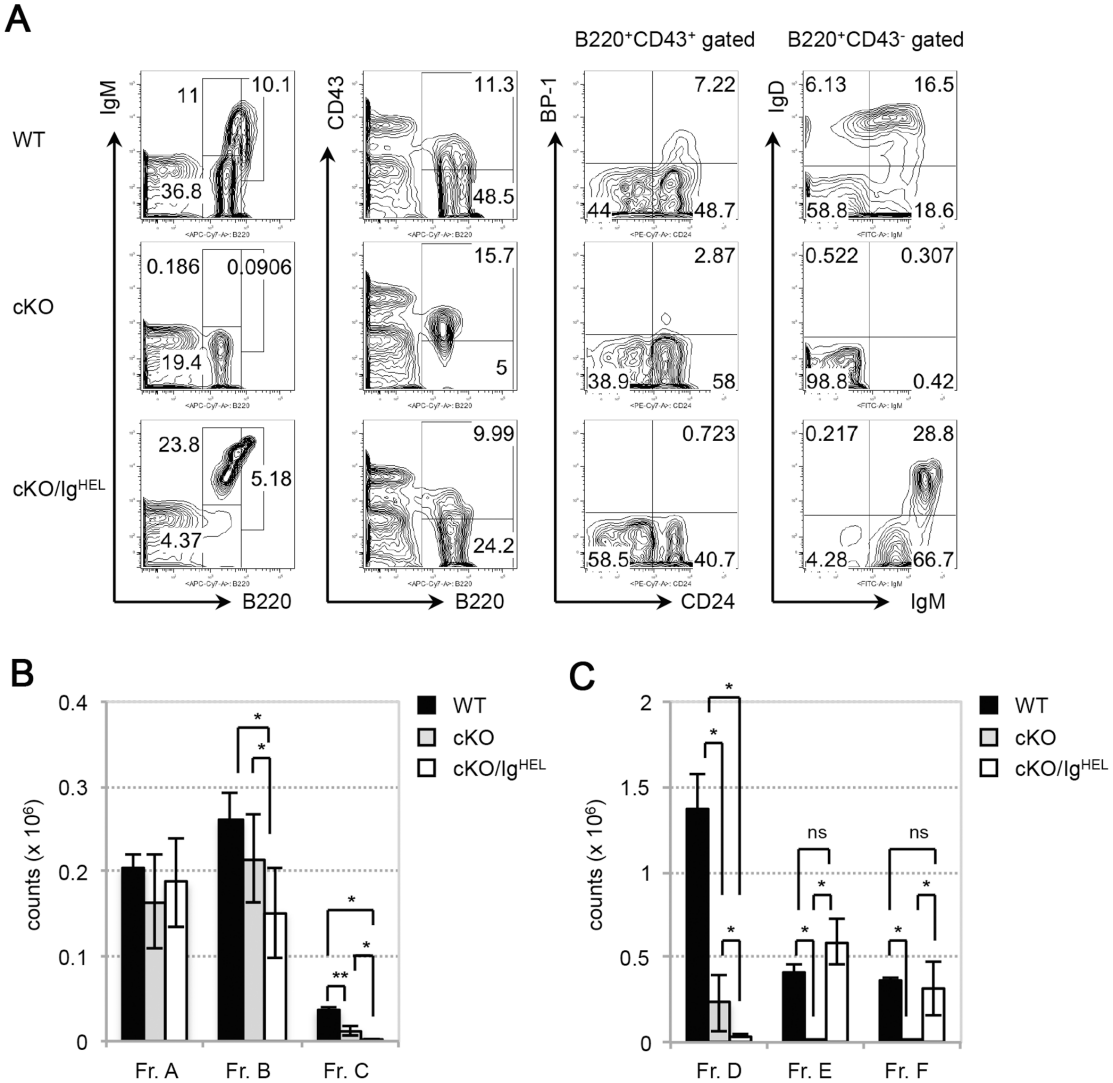
The defect in B cell development caused by PP4 deficiency can be rescued by Ig transgene expression. (A) Flow cytometric profiles of: total BM lymphocytes from WT, cKO and cKO/Ig^HEL^ mice (n = 3–5/group) analyzed for B220 vs IgM (far left panel) or B220 vs CD43 (middle left panel); or B220^+^CD43^+^ BM lymphocytes analyzed for CD24 vs BP-1 (middle right panel); or B220^+^CD43^−^ BM lymphocytes analyzed for IgM vs IgD (far right panel). (B) Quantitative cell count analysis of Frs. A to C cells from the mice in (A). (C) Quantitative cell count analysis of Frs. D to F cells from the mice in (A). For B and C, data are the mean ± SD (n = 3–5/group). Results are representative of two independent experiments (*p≤0.05, **p≤0.005; ns, not significant).

Based on our findings above, we hypothesized that PP4 is required for pro-B cell development because it promotes Ig DJ_H_/V_H_DJ_H_ recombination. To test this theory, we crossed our cKO mice to Ig^HEL^ transgenic mice [32] to generate cKO/Ig^HEL^ mice. The Ig^HEL^ transgene drives constitutive BCR expression, which should overcome the developmental block in PP4-deficient pro-B cells if this hurdle is caused by impaired Ig DJ_H_/V_H_DJ_H_ recombination [38]. B cell subsets from the BM of cKO/Ig^HEL^ mice were analyzed and compared to those of WT and cKO mice. Interestingly, although numbers of Fr. C and Fr. D cells in cKO/Ig^HEL^ mice were not restored to WT levels (Fig. 5A, 5B), B220^+^IgM^+^ immature B cells and B220^high^IgM^+^ B cells became detectable by FACS analysis (Fig. 5A, left panel). By gating on B220^+^CD43^−^ lymphocytes, we were able to discern that the percentages of IgM^+^IgD^−^ cells (Fr. E) and IgM^+^IgD^+^ cells (Fr. F) in cKO/Ig^HEL^ mice had increased to 66.7% and 28.8% (Fig. 5A, right panel), respectively, which were higher than those in cKO mice. Furthermore, cell counts of Fr. E and Fr. F cells in cKO/Ig^HEL^ mice reached WT levels, indicating that the Ig transgene was able to circumvent the developmental arrest imposed by PP4 deficiency (Fig. 5C). It has been previously reported that Ig transgenic mice show an acceleration in B cell development prior to the Fr. E stage [39]. Thus, the near-absence of Fr. C and Fr. D cells in cKO/Ig^HEL^ mice was likely mediated by their expression of the Ig transgene. We can therefore conclude that the Ig transgene was able to drive PP4-deficient B cell development by promoting the differentiation of IgM^+^ B cells.

## Discussion

In this study, we describe the essential role of PP4 in early B cell development through its regulation of Ig DJ_H_/V_H_DJ_H_ recombination. Utilizing the mb-1/cre-flox system to generate cKO mice, we have shown that cre activity under the control of the mb-1 promoter initiates at the pre-pro-B cell stage (Fr. A). However, the deletion efficiency of the *pp4c* allele in Fr. A cells was poor, precluding us from investigating the function of PP4 in this population. Fortunately, acceptable deletion efficiencies were achieved in cKO Fr. B and Fr. C cells, permitting us to determine the effects of PP4 deficiency on these subsets. With respect to Fr. B cells, loss of PP4 leads to impaired Ig DJ_H_ recombination accompanied by reduced µ HC expression. These defects occur in the absence of abnormal cell-cycle progression or excessive apoptosis. During WT B cell development, secondary DJ_H_ rearrangements, which employ a more 5′ D segment to replace the existing DJ_H_ rearrangement, occur frequently in pro-B and pre-B cells [40]. Hence, it is likely that PP4-deficient Fr. B cells, which fail to complete DJ_H_/V_H_DJ_H_ recombination, undergo a secondary DJ_H_/V_H_DJ_H_ rearrangement rather than apoptosis. Consistent with that, we found that PP4-deficeint Fr. B cells generate more coding ends at 3′ RSS sites of DFL16.1 and DSP. It suggests that the impaired DJ_H_ recombination by PP4 deficiency increases the usage of more 5′ D segments for a secondary rearrangement. Very importantly, the analyses of comet scores and apoptosis in Fr. B cells (shown in Fig. 4A and 4F) did not show any significant difference between WT and cKO mice. The data strongly suggest that PP4 regulates Ig DJ_H_/V_H_DJ_H_ recombination at the joining phase such that loss of *pp4c* causes DSBs at Ig loci.

In contrast to Fr. B cells, cells in Fr. C that fail to accomplish a secondary or tertiary DJ_H_/V_H_DJ_H_ rearrangement do undergo apoptosis. It has been proposed that such premature death is invoked to prevent the survival of a cell with oncogenic potential [41]. This hypothesis might explain why WT Fr. C cells display more apoptosis than WT Fr. B cells. Even so, apoptosis in cKO Fr. C cells is more severe than in WT Fr. C cells, consistent with the drastic reduction in Fr. C cell counts in the mutant mouse. Because the sole defect in DNA repair during V(D)J recombination would not lead to the formation of visible comets, it is likely that the severe DNA fragmentation in PP4-deficient Fr. C cells is due to other mechanisms, such as apoptosis [42]. Nevertheless, cKO Fr. C cells were observed to accumulate in the G1 phase, indicating a disruption of cell-cycle progression. It is noteworthy that V_H_DJ_H_ recombination is normally restricted at the G1 phase to ensure the repair of recombination-liberated DNA ends prior to re-entry of the cell-cycle [33]. The accumulation of G1 phase Fr. C cells in cKO mice, together with the strong induction of coding ends-associated DSBs in Fr. B cells, likely reflects the fact that Fr. C cells lacking PP4 cannot complete V_H_DJ_H_ rearrangement and thus undergo apoptosis. Consequently, PP4 deficiency results in a severe reduction of Fr. C cells and developmental arrest. We were able to restore IgM^+^ BM B cells to WT levels in PP4-deficient mice through transgenic Ig expression. This finding strongly supports our hypothesis that PP4 is indispensable for Ig V_H_DJ_H_ recombination. It also reveals that the transgenic Ig transmitted functional BCR signaling that overcame the PP4 deficiency and supported the differentiation of IgM^+^ B cells.

Intriguingly, the phenotype of mice bearing a B cell-specific deletion of PP4 is more drastic than the block in T cell development exhibited by mice bearing a Lck-cre-mediated T cell-specific deletion of PP4, as CD4^+^ and CD8^+^ single positive T cells are present in both the thymus and periphery of these mutants [25]. During T cell development, DN2 and DN3 thymocytes, which are undergoing TCRß gene rearrangement, are the cells equivalent to the developing Fr. B cells. Because the deletion efficiencies in DN2 and DN3 thymocytes from those mutant mice were not reported, it was not clear whether cre-affiliated leakiness could result in a less severe development block than the effect of mb-1/cre mediated PP4 deficiency in B cells. Although the mechanism for VDJ recombination is in general highly conserved between B and T lymphocytes, gene-targeting of NHEJ factors in some cases can lead to unequal severities in B and T cell developments. For example, in Ku70 knockout (KO) mice and DNA polymerase µ (Pol µ) KO mice, VDJ recombination at Ig loci is drastically impaired, whereas TCRß/α gene rearrangement is functional [43] [44] [45]. A comprehensive recombination assay in PP4-deficient thymocytes would be necessary to address whether PP4 is also involved in TCR gene rearrangement.

PP4 has been implicated in two DNA repair pathways: homologous recombination-mediated repair of DNA breaks via dephosphorylation of γH2AX [18], [24], and NHEJ-mediated end-joining by dephosphorylation of KAP1 (KRAB-associated protein 1) [46]. Whether the disruption of γH2AX or KAP1 phosphorylation status can lead to abrupt developmental arrest at the pro-B cell stage requires further investigation. In any case, the results of our study provide the first genetic evidence establishing a critical role for PP4 in pro-B cell development through its promotion of Ig V_H_DJ_H_ recombination.

## Supporting Information

Figure S1 Schematic diagram of southern blotting strategy used to detect DJ_H_ recombination.
(TIFF)

Figure S2 Schematic diagram of southern blotting strategy used to detect coding ends at 3′ of D segments.
(TIFF)
