# Supplementary figures and images for: The Serine/Threonine Phosphatase PP4 Is Required for Pro-B Cell Development through Its Promotion of Immunoglobulin VDJ Recombination
Source: PLoS One. 2013 Jul 16;8(7):e68804. doi: 10.1371/journal.pone.0068804 (PMC3712940; doi:10.1371/journal.pone.0068804)

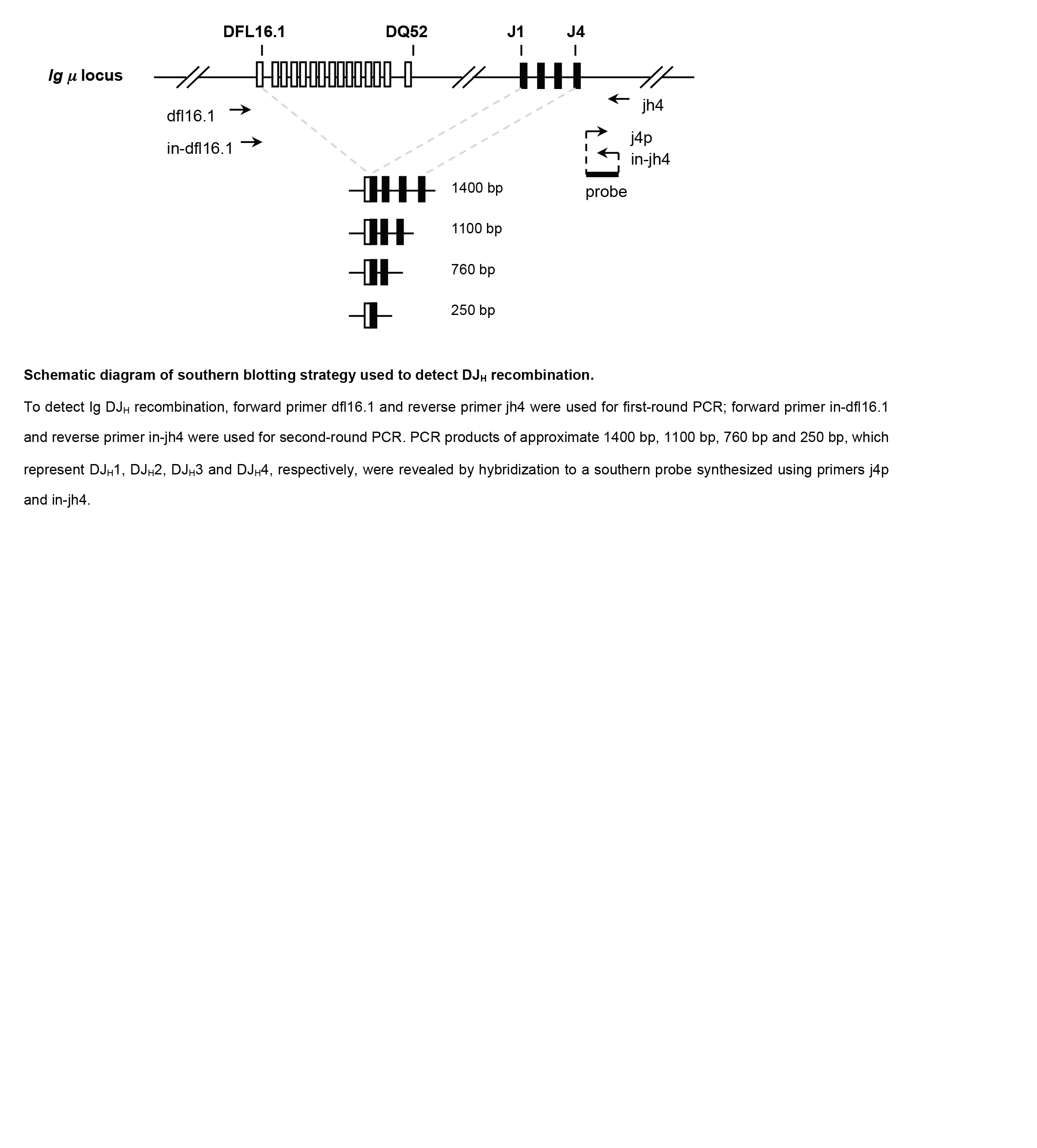

Supplement: Figure S1 — Schematic diagram of southern blotting strategy used to detect DJH recombination. (TIFF) [file pone.0068804.s001.tiff]

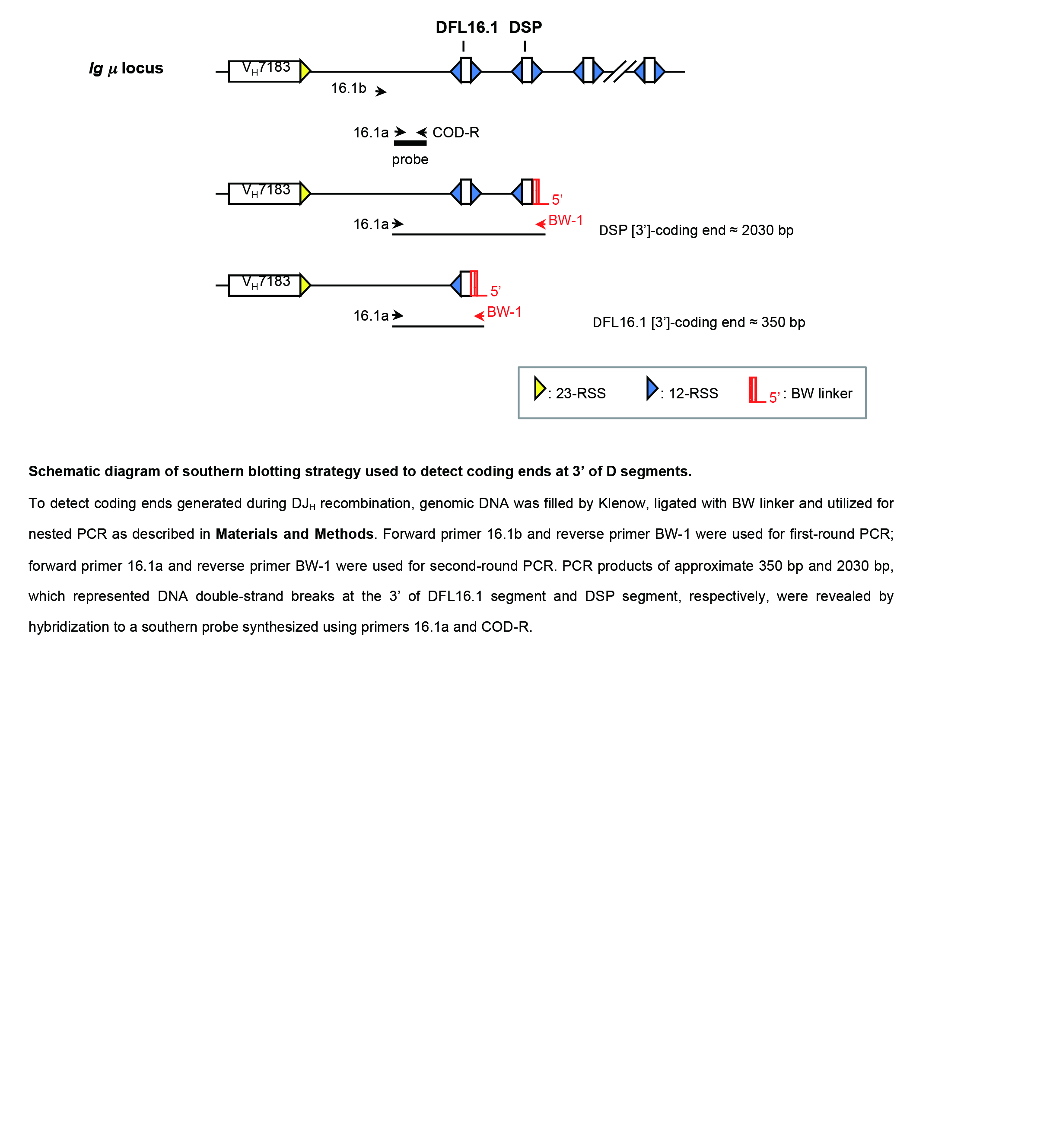

Supplement: Figure S2 — Schematic diagram of southern blotting strategy used to detect coding ends at 3′ of D segments. (TIFF) [file pone.0068804.s002.tiff]
